# Protective effects of *Brassica rapa* L. water extract against DSS-induced acute UC-like colitis in mice and its regulatory mechanisms involving gut microbiota and the intestinal mucosal barrier

**DOI:** 10.3389/fmicb.2026.1829670

**Published:** 2026-07-13

**Authors:** Jiaying Wu, Xiuyingzi Zhu, Xuwen Mao

**Affiliations:** 1 College of Pharmacy, Xinjiang Medical University, Urumqi, China; 2 Xinjiang Key Laboratory of Biopharmaceuticals and Medical Devices, Xinjiang Medical University, Urumqi, China

**Keywords:** Acute colitis-like injury, *Brassica rapa* L. water extract, DSS-induced acute UC-like colitis, gut microbiota, intestinal mucosal barrier, metabolomics

## Abstract

**Introduction:**

The present study aimed to evaluate the protective effects of *Brassica rapa* L. (Br) water extract against dextran sulfate sodium (DSS)-induced acute ulcerative colitis (UC)-like injury in mice and to explore the underlying mechanisms.

**Methods:**

A DSS-induced acute UC-like colitis mouse model was established to assess the effects of Br administration. Disease severity was evaluated based on body weight loss, disease activity index (DAI) scores, colon length, colon weight, intestinal barrier integrity, and histopathological changes using H&E staining. Inflammatory mediators, TLR4/TLR9 expression, tight junction (TJ) proteins, gut microbiota composition by 16S rRNA sequencing, and intestinal metabolic profiles by UPLC-MS/MS-based non-targeted metabolomics were also analyzed.

**Results:**

Br administration significantly ameliorated disease severity, as evidenced by mitigated body weight loss, reduced disease activity index (DAI) scores, attenuated colon shortening, normalized colon weight, and improved intestinal barrier integrity. Histopathological analysis (H&E) demonstrated that Br alleviated colonic tissue damage and inflammatory cell infiltration. The treatment also decreased the levels of proinflammatory mediators (IL-6, MPO, CXCL-1, and TNF-α) and downregulated the colonic expression of TLR4 and TLR9, while upregulating the expression of tight junction (TJ) proteins Occludin (OCC) and ZO-1. Furthermore, 16S rRNA sequencing revealed that Br restored gut microbial diversity and composition, specifically enriching potentially beneficial genera such as *Alloprevotella, Lachnospiraceae_unclassified*, and *Alistipes*, while suppressing the expansion of genera such as *HT002*. UPLC-MS/MS-based non-targeted metabolomic analysis suggested that Br was associated with the modulation of intestinal metabolic profiles, including changes in several putatively annotated metabolite features such as 12-Hydroxy-12-octadecanoylcarnitine and Vanillin.

**Discussion:**

Br water extract alleviates DSS-induced acute UC-like colitis injury in mice, possibly by attenuating inflammatory responses, downregulating abnormal TLR4/TLR9 expression, improving intestinal mucosal barrier function, and modulating gut microbiota composition and associated metabolic profiles.

## 1 Introduction

Inflammatory bowel disease (IBD), mainly including ulcerative colitis (UC) and Crohn's disease (CD), is a chronic, relapsing inflammatory disorder of the gastrointestinal tract (Corridoni et al., 2014). In recent years, the global incidence of IBD has continued to rise, and the disease has become a common digestive disorder and a significant health burden in China (Yu et al., 2024). Among the two major subtypes of IBD, UC is characterized by continuous mucosal inflammation that primarily affects the colon and rectum, and its clinical course is often prolonged and recurrent. The difficulty of achieving sustained remission has a considerable negative impact on patients' quality of life. Currently, treatment for UC remains primarily based on amino salicylates, corticosteroids, immune suppressants, and biologics; however, these therapies are limited by significant inter individual variability in efficacy, multiple long-term side effects, and high recurrence rates after discontinuation.

Research indicates that UC is not exclusively attributable to immunological abnormalities but arises from the interplay of genetic susceptibility, gut microbiota dysbiosis, intestinal mucosal barrier dysfunction, and aberrant innate immune recognition (Ramos and Papadakis, 2019; Neurath, 2020; Ni et al., 2017; Lloyd-Price et al., 2019). Patients with UC frequently demonstrate diminished gut microbiota diversity, reduced abundance of obligate anaerobes and beneficial bacteria associated with short-chain fatty acid production, alongside aberrant proliferation of facultative anaerobes and potential pathogens. These alterations are closely associated with disease activity, recurrence risk, and treatment response (Haiser et al., 2019; Yilmaz et al., 2019). Beyond dysbiosis, UC is also accompanied by marked alterations in the gut metabolic profile (Li et al., 2022; Cai et al., 2022). Microbiota-associated metabolites, such as bile acids and short-chain fatty acids, undergo significant alterations in UC, thereby further influencing mucosal immune homeostasis, epithelial energy metabolism, and inflammatory responses (Parada Venegas et al., 2019; Bromke and Krzystek-Korpacka, 2021; Burgueño and Abreu, 2020). Concurrently, impaired intestinal mucosal barrier function represents a critical component of UC progression. Downregulation of tight junction proteins increases epithelial permeability, facilitating the entry of bacterial products and nucleic acids into the mucosa and activating innate immune responses (Vancamelbeke and Vermeire, 2017; Turner, 2009). Therefore, comprehensive research focusing on gut microbiota structure, metabolic profile remodeling, and mucosal barrier damage may contribute to a more thorough understanding of UC pathogenesis and provide novel insights for intervention strategies.

In recent years, traditional Chinese medicine (TCM) therapies have garnered significant attention for the treatment of UC because of their anti-inflammatory, immunomodulatory, and antioxidant properties (Yuan et al., 2022a,b). *Brassica rapa* L. (Br), a distinctive medicinal and edible plant native to Xinjiang, consists of the dried mature seeds of the cruciferous plant turnip. Modern research indicates that it is rich in glucosinolates, isothiocyanates, phenolic compounds, polysaccharides, and organic acids (Paul et al., 2019; Sousa et al., 2008). Notably, polysaccharides derived from Br exert gut-protective effects by modulating the gut microbiota and their metabolites, suppressing inflammatory responses, and enhancing intestinal barrier function (Liu et al., 2022, 2024). In addition, indole components in Br inhibit the NF-κB signaling pathway and downstream inflammatory mediators (Niu et al., 2024; Cho et al., 2012), further supporting its potential value for UC-related intestinal inflammation. However, studies investigating whether Br aqueous extract can alleviate DSS-induced acute UC-like colitis by regulating gut microbiota and metabolic profiles remain scarce. DSS-induced acute colitis is widely used to mimic several pathological features of UC, particularly colonic epithelial injury, mucosal inflammation, intestinal barrier disruption, and colon shortening. Therefore, this study employed a DSS-induced acute UC-like colitis model and a comprehensive approach integrating histopathology, inflammatory cytokine analysis, immunohistochemical analysis of TLR4/TLR9 and tight junction proteins, 16S rRNA sequencing, and non-targeted metabolomics to systematically evaluate the protective effects of Br and its potential mechanisms.

## 2 Materials and methods

### 2.1 Materials and reagents

ZO-1 Rabbit pAb and Occludin Rabbit pAb were purchased from Thermo Fisher Scientific. TLR4 Rabbit pAb was purchased from Novus Biologicals. TLR9 Rabbit pAb was purchased from Bioss Antibodies. Dextran sulfate sodium salt (DSS) was purchased from Shanghai Kelin Biochemical Technology Co., Ltd., batch No. c12750877. Dexamethasone injection was purchased from Shanghai Modern Hassen (Shangqiu) Pharmaceutical Co., Ltd., batch No. 1905190231.

### 2.2 Preparation of *Brassica rapa* L. water extract

*Brassica rapa* L. (Br) was purchased from Xinjiang Ailinuoer Chamagou Industrial Biotechnology Co., Ltd. Ten grams of Br powder were weighed, mixed with distilled water, and refluxed at 80 °C for 1 h. Following three extractions, the extracts were amalgamated and condensed to yield a dry extract with a crude drug content of 1 g/mL. The dry extract was subsequently dissolved in water to create an aqueous solution with a concentration of 2 mg/mL. The aqueous extract of Br contains a relatively high level of phenolic compounds.

### 2.3 Establishment of DSS-induced acute UC-like colitis model and drug intervention

Sixty male C57BL/6J mice, aged 6–8 weeks and weighing 20–24 g, were purchased from the Animal Experiment Center of Xinjiang Medical University. All mice were housed under controlled conditions at a temperature of 21 ± 2 °C, a relative humidity of 40–45%, and a 12-h light/dark cycle.

Mice were randomly divided into six groups (n = 10): Control, DSS, DEX, and three DSS+Br treatment groups, namely Br-L, Br-M, and Br-H. The Control group received normal drinking water and was administered sterile water by gavage once daily. Except for the Control group, all other groups were provided with unrestricted access to 3% DSS solution for 10 consecutive days to induce acute UC-like colitis. The DSS group was administered sterile water by gavage once daily as the vehicle control. The DEX group was treated with dexamethasone at a dose of 0.4 mg/kg by intraperitoneal injection. The DSS + Br groups received Br water extract by gavage once daily at doses of 25, 50, and 100 mg/kg for the Br-L, Br-M, and Br-H groups, respectively. Body weight, fecal characteristics, and rectal bleeding were recorded daily, and disease activity index (DAI) scores were calculated to evaluate colitis severity. On day 11, mice were euthanized by intraperitoneal injection of sodium pentobarbital (100–150 mg/kg). Colon tissues and fresh fecal samples were collected for subsequent analyses.

### 2.4 Intestinal permeability assessment

After a 4-h fast, mice in each group were orally administered fluorescein isothiocyanate-dextran (FITC-dextran; 1 mg/mL) by gavage. The FITC-dextran solution was freshly prepared and protected from light before use. Three h after gavage, blood samples were collected from the orbital vein and centrifuged to obtain serum. Serum FITC-dextran levels were detected at 490 nm using a microplate reader. FITC-dextran concentrations were calculated according to a standard curve prepared with known concentrations of FITC-dextran. Intestinal permeability was evaluated based on the serum FITC-dextran concentration.

### 2.5 Hematoxylin and eosin staining

Colon tissues were fixed in 4% paraformaldehyde, dehydrated, embedded in paraffin, and sectioned at 5 μm. The sections were deparaffinized, rehydrated, stained with hematoxylin and eosin, dehydrated, cleared, and mounted. Histopathological changes, including epithelial injury, crypt architecture disruption, mucosal edema, and inflammatory cell infiltration, were observed under a light microscope. Histological injury was evaluated in a blinded manner using a semi-quantitative scoring system. The scoring criteria included inflammatory cell infiltration, inflammation depth, crypt/epithelial damage, and lesion extent, with a total score ranging from 0 to 14. Higher scores indicated more severe colonic injury. Multiple randomly selected fields from each mouse were evaluated, and the average score was used for statistical analysis.

### 2.6 ELISA analysis

Commercial ELISA kits were used to measure the concentrations of IL-6, MPO, CXCL-1, and TNF-α in mouse serum according to the manufacturers' instructions. Blood samples were collected, centrifuged, and stored at −80 °C. Standards and samples were added to the pre-coated plates. Antibody incubation and TMB color development were performed sequentially according to the kit protocol. Absorbance was measured at 450 nm, and cytokine concentrations were calculated accordingly.

### 2.7 Immunohistochemistry

Colon tissues were fixed, embedded in paraffin, and sectioned at a thickness of approximately 5 μm. The sections were deparaffinized in xylene and rehydrated through a graded ethanol series. Antigen retrieval was performed by heating the sections in citrate buffer, followed by cooling to room temperature. Endogenous peroxidase activity was blocked, and non-specific binding was blocked with normal serum. The sections were then incubated overnight at 4 °C with the following primary antibodies: anti-ZO-1 rabbit polyclonal antibody, anti-Occludin rabbit polyclonal antibody, anti-TLR4 rabbit polyclonal antibody, and anti-TLR9 rabbit polyclonal antibody. After washing with PBS, the sections were incubated with an HRP-conjugated goat anti-rabbit IgG secondary antibody at room temperature. DAB was used for chromogenic development, followed by hematoxylin counterstaining. The sections were then dehydrated, cleared, and mounted with neutral resin. Images were captured under a light microscope and semi-quantitatively analyzed using ImageJ software. The expression level of each target protein was assessed based on the percentage of positively stained area.

### 2.8 Gut microbiota sequencing

Fecal samples were suspended in preheated CTAB extraction buffer (65 °C) and incubated for 60 min with intermittent mixing to ensure thorough lysis. The lysate was centrifuged at 8,000 rpm for 5 min, and the supernatant was sequentially extracted twice with chloroform: isoamyl alcohol (24:1, v/v) to remove proteins and other contaminants. DNA was precipitated by adding isopropanol and sodium acetate, followed by centrifugation, air-drying, and resuspension in sterile deionized water containing RNase A (10 mg mL^−1^). After incubation at 37 °C for 1 h to digest RNA, the purified DNA was stored at −20 °C. The CTAB extraction buffer consisted of 2% (w/v) CTAB, 1.4 M NaCl, 100 mM Tris-HCl (pH 8.0), and 25 mM EDTA, with 0.2% (v/v) β-mercaptoethanol added prior to use. Subsequently, DNA concentration was quantified using a UV spectrophotometer, and 50 ng of template DNA was used for PCR amplification. Library quality was assessed using an Agilent 2100 Bioanalyzer, and library quantification was performed using the Illumina library quantification kit prior to paired-end sequencing. Sequencing data were demultiplexed, and adapter and barcode sequences were removed. Sequences were then subjected to assembly and quality filtering, and the DADA2 denoise-paired module on the QIIME2 platform was used for sequence length trimming and denoising, generating ASV representative sequences and abundance tables.

### 2.9 Bioinformatics data analysis

Microbial α-diversity was assessed based on the obtained ASVs and their abundance matrices. Taxonomic annotation was performed using the SILVA database with a confidence threshold of 0.7. For comparisons between two groups with biological replicates, the Mann–Whitney U test was used. For comparisons among multiple groups, the Kruskal–Wallis test was used. Correlation analysis of the microbial community was performed using the Microbiology Alliance platform (Tang et al., 2023), while community composition analysis and the corresponding heat maps were generated using the Microbiome Informatics platform (Gao et al., 2024).

### 2.10 UPLC-MS/MS non-targeted metabolomics analysis

Fecal samples were thawed on ice, and 100 mg of each sample was weighed and transferred to a pre-chilled centrifuge tube. Cold methanol extraction solvent and steel balls were added, and metabolites were extracted using combined homogenization and cold ultrasonication. The extract was centrifuged at high speed at 4 °C, and the supernatant was collected for metabolomic analysis. For quality control, QC samples were prepared by mixing equal volumes of extract from each sample and were inserted throughout the detection sequence. Solvent blanks were included to monitor the system background. Samples were analyzed using UPLC-MS/MS with full-scan acquisition in both positive and negative ion modes. The acquired data were used for subsequent metabolite peak identification, data alignment, and statistical analysis.

### 2.11 Statistical analysis

The experimental results in this study are expressed as the mean ± standard deviation (SD). Data analysis and graphical representation were predominantly conducted using GraphPad Prism 9.0 software. Unpaired *t*-tests were used to compare two groups of data that satisfied the normal distribution assumption. One-way analysis of variance (ANOVA) was used to evaluate overall statistical significance when comparing three or more groups. *P* < 0.05 was considered statistically significant.

## 3 Result

### 3.1 Br alleviates DSS-induced acute UC-like colitis injury and suppresses inflammatory responses

**Figure 1:**
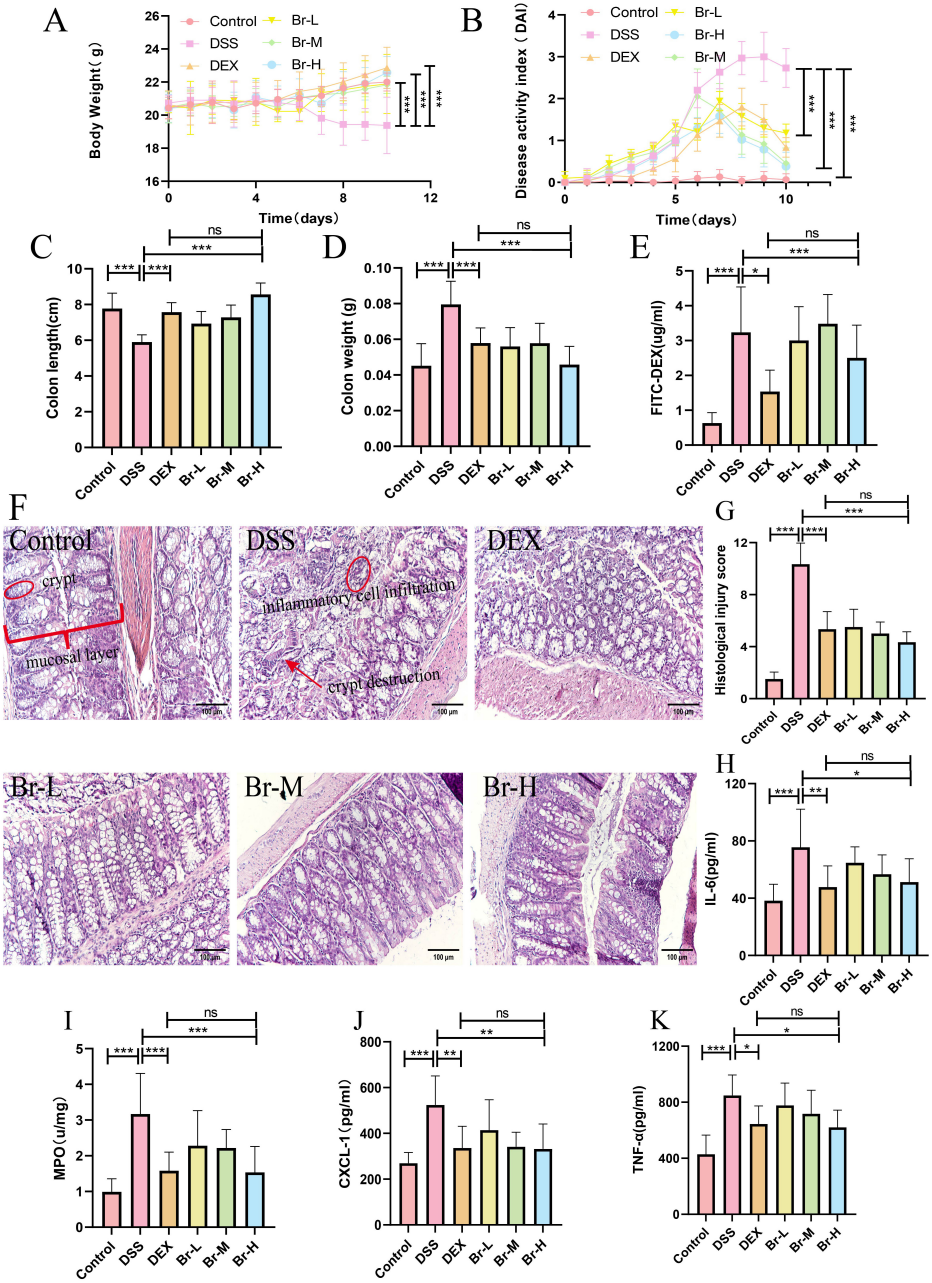
Br ameliorates DSS-induced acute UC-like colitis symptoms in mice (*n* = 10). **(A)** Body weight; **(B)** disease activity index (DAI) score; **(C)** colon length; **(D)** colon weight; **(E)** intestinal permeability assessed by FITC-dextran; **(F)** hematoxylin and eosin (H&E) staining of colonic tissues (*n* = 6). In the Control panel, the red ellipse indicates representative colonic crypts, and the red bracket indicates the colonic mucosal layer. In the DSS panel, arrows indicate crypt structure disruption, and the red ellipse indicates inflammatory cell infiltration. Scale bar = 100 μm. **(G)** Histological injury scores of colon tissues based on inflammatory cell infiltration, inflammation depth, crypt/epithelial damage, and lesion extent; **(H**) concentrations of IL-6; **(I)** concentrations of MPO; **(J)** concentrations of CXCL-1; **(K)** concentrations of TNF-α. ^***^P < 0.001, ^**^P < 0.01, ^*^P < 0.05; ns indicates no significant difference. Data are expressed as the mean ± SD.

Compared with the Control group, DSS-treated mice showed significant body weight loss, increased DAI scores, shortened colon length, elevated colon weight, and increased intestinal permeability, indicating the successful establishment of DSS-induced acute UC-like colitis (*P* < 0.05). Compared with the DSS group, DEX significantly improved these disease-related parameters, confirming its protective effect as a positive control. Br treatment also alleviated DSS-induced acute UC-like colitis in a dose-dependent manner, with Br-H showing the most pronounced improvement among the Br-treated groups, as reflected by restored body weight, reduced DAI scores, improved colon length, decreased colon weight, and reduced FITC-dextran levels (*P* < 0.05). The overall protective effect of Br-H was comparable to that of DEX for these disease-related parameters (Figures 1A–E). Histopathological examination showed that DSS treatment caused severe colonic injury, including crypt atrophy, mucosal architecture disruption, mucosal edema, and inflammatory cell infiltration. DEX and Br treatment alleviated these pathological alterations, with Br-H showing the most evident improvement among the Br-treated groups and a protective effect comparable to that of DEX (Figure 1F). Histological injury scoring showed that DSS significantly increased colonic injury scores, whereas DEX and Br treatment reduced the scores to varying degrees (*P* < 0.05; Figure 1G). In addition, DSS exposure significantly increased the levels of proinflammatory mediators, including IL-6, MPO, CXCL-1, and TNF-α (*P* < 0.05). Compared with the DSS group, DEX significantly reduced these inflammatory mediators (*P* < 0.05). Br administration also suppressed the DSS-induced inflammatory response in a dose-dependent manner. Among the Br-treated groups, Br-H showed the strongest reduction in IL-6, MPO, CXCL-1, and TNF-α levels (*P* < 0.05), with an anti-inflammatory effect comparable to that of DEX (Figures 1H–K). These results indicate that Br, especially at the high dose, attenuates DSS-induced acute UC-like colitis, improves intestinal barrier function, and suppresses inflammatory responses, with effects comparable to those of DEX.

### 3.2 Br inhibits inflammation-related protein expression and improves intestinal mucosal barrier function in DSS-induced acute UC-like colitis

**Figure 2:**
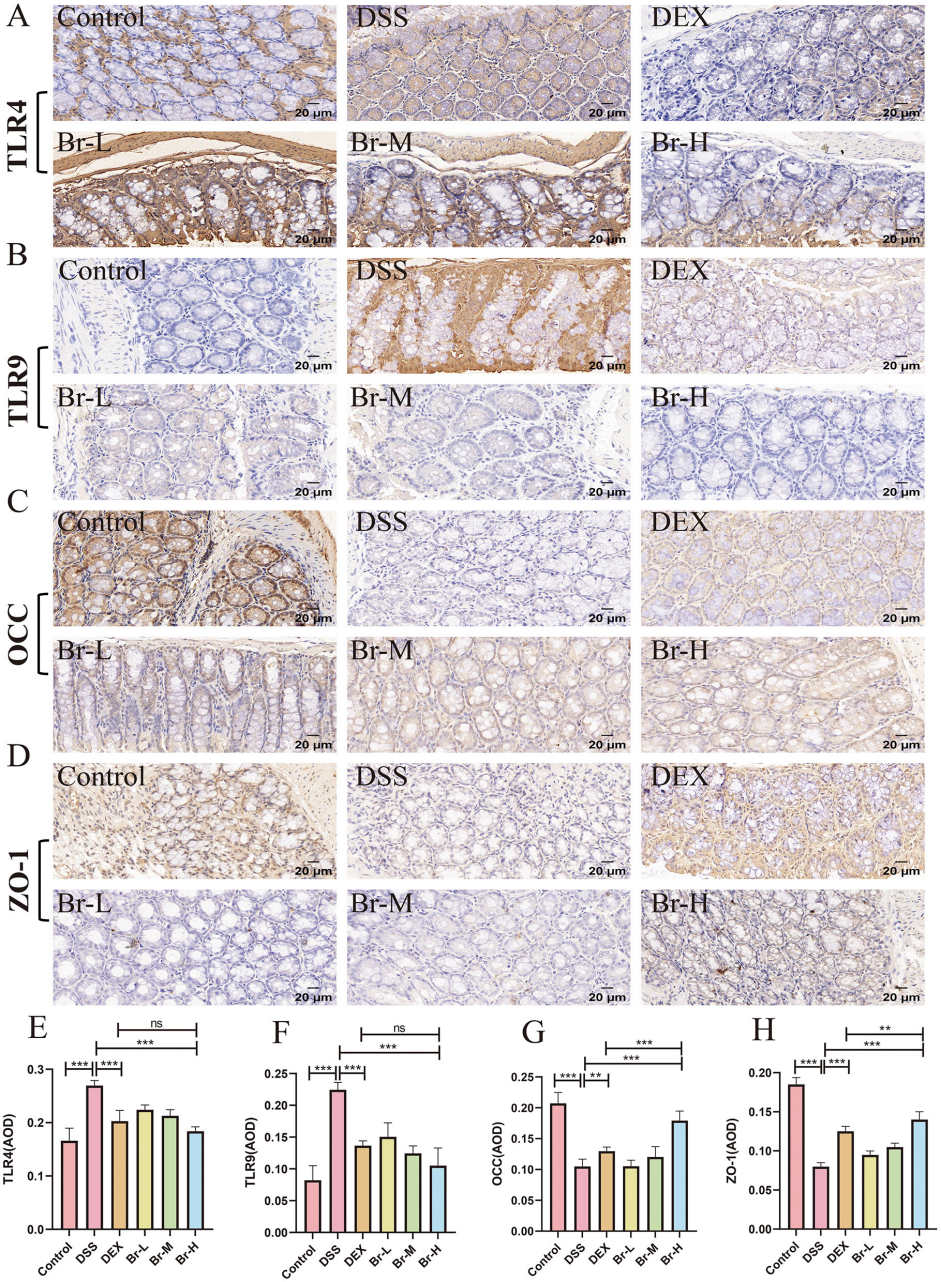
Br suppresses inflammation-related protein expression and restores intestinal mucosal barrier function in DSS-induced acute UC-like colitis mice (*n* = 6). **(A)** Representative images of colonic TLR4 immunohistochemical staining; **(B)** Representative images of colonic TLR9 immunohistochemical staining; **(C)** Representative images of colonic OCC immunohistochemical staining; **(D)** Representative images of colonic ZO-1 immunohistochemical staining; **(E)** Semi-quantitative analysis of the average optical density (AOD) of TLR4; **(F)** Semi-quantitative analysis of the average optical density (AOD) of TLR9; **(G)** Semi-quantitative analysis of the average optical density (AOD) of OCC; **(H)** Semi-quantitative analysis of the average optical density (AOD) of ZO-1. ^***^P < 0.001, ^**^P < 0.01, ^*^P < 0.05, ns indicates no significant difference. Data are expressed as the mean ± SD.

TLR4 and TLR9 are closely associated with colonic inflammatory responses, whereas OCC and ZO-1 are key tight junction proteins involved in maintaining intestinal epithelial barrier integrity. Immuno histochemical staining showed brownish-yellow positive signals for these proteins in colonic tissues (Figures 2A–D). Compared with the Control group, DSS treatment significantly increased the expression of TLR4 and TLR9, while markedly decreasing the expression of OCC and ZO-1, indicating enhanced inflammatory activation and impaired mucosal barrier function (*P* < 0.05). Compared with the DSS group, DEX significantly suppressed TLR4 and TLR9 expression and restored OCC and ZO-1 expression, confirming its protective effect as a positive control (*P* < 0.05). Similarly, Br treatment reversed these DSS-induced changes to varying degrees, with Br-H showing the most pronounced regulatory effect on inflammation-related proteins and tight junction proteins among the Br-treated groups (*P* < 0.05). The regulatory effect of Br-H was comparable to that observed in the DEX group (Figures 2E–H). These results suggest that Br-H may improve intestinal mucosal barrier function in DSS-induced acute UC-like colitis by reducing inflammation-related protein expression and restoring tight junction protein levels, with effects comparable to those of DEX.

### 3.3 Br improves gut microbiota diversity, richness, and community structure in mice with DSS-induced acute UC-like colitis

**Figure 3:**
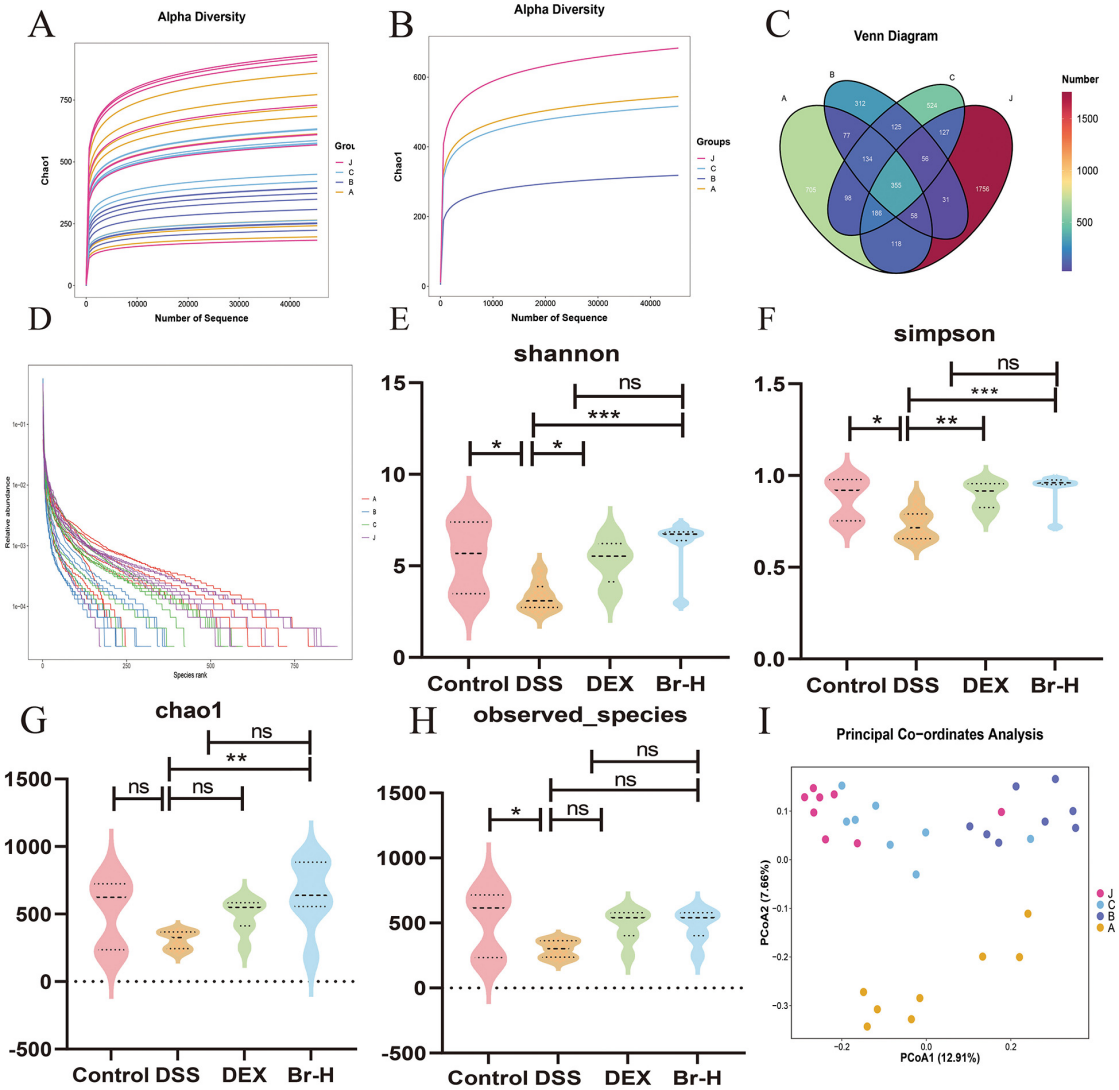
Br improves the diversity and richness of the gut microbiota in mice with DSS-induced acute UC-like colitis (*n* = 8). **(A, B)** Rarefaction curves of the Chao1 index; **(C)** Venn diagram of ASVs among groups; **(D)** Rank-abundance curves of the microbial communities; **(E**) Shannon index; **(F)** Simpson index; **(G)** Chao1 index; **(H)** Observed species index; **(I)** Principal co-ordinates analysis (PCoA) plot showing β-diversity and overall differences in gut microbial community structure among groups. ^***^P < 0.001, ^**^P < 0.01, ^*^P < 0.05; ns indicates no significant difference. Data are expressed as mean ± SD. In panel I, A, B, C, and J represent the Control, DSS, DEX, and Br-H groups, respectively.

The rarefaction curves gradually flattened, indicating that the sequencing depth was sufficient to capture most microbial species and support subsequent diversity analysis (Figures 3A, B). ASV analysis identified 705, 312, 524, and 1,756 ASVs in the Control, DSS, DEX, and Br-H groups, respectively. Compared with the Control group, the DSS group showed a marked reduction in ASV numbers, indicating decreased microbial richness. Compared with the DSS group, both DEX and Br-H increased ASV numbers, with Br-H showing the highest ASV abundance among all groups (Figure 3C). Rank-abundance curves further showed that microbial richness was reduced after DSS treatment, whereas DEX and Br-H partially restored species richness, especially in the Br-H group (Figure 3D). α-diversity analysis showed that DSS significantly decreased the Shannon, Simpson, and observed-species indices compared with the Control group (*P* < 0.05), accompanied by a decreasing trend in the Chao1 index. Compared with the DSS group, DEX and Br-H improved these α-diversity indices to varying degrees, with Br-H generally approaching the Control level (Figures 3E–H). β-diversity analysis based on principal coordinate analysis (PCoA) further showed that the DSS group tended to separate from the Control group, suggesting that DSS treatment altered the overall gut microbial community structure. DEX and Br-H treatment changed the distribution pattern of DSS-treated samples to varying degrees, indicating partial modulation of DSS-induced microbial dysbiosis (Figure 3I). These results indicate that Br-H improves gut microbiota richness and diversity and partially reshapes the overall microbial community structure in mice with DSS-induced acute UC-like colitis.

### 3.4 Br modulates gut microbiota composition in mice with DSS-induced acute UC-like colitis

**Figure 4:**
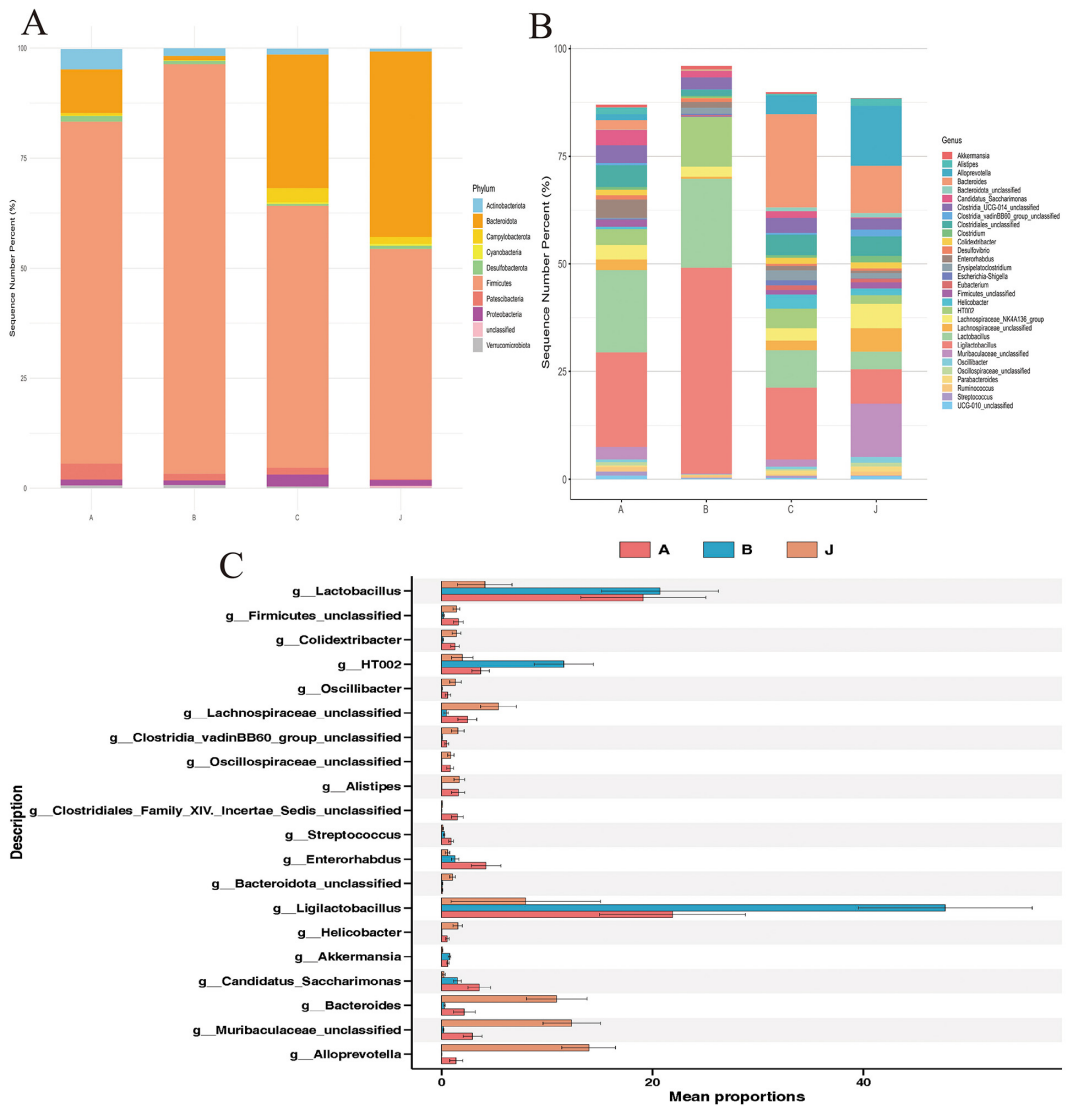
Br modulates the composition of the gut microbiota in mice with DSS-induced acute UC-like colitis (*n* = 8). **(A)** Phylum-level composition of the intestinal microbiota; **(B)** Genus-level composition of the intestinal microbiota; **(C)** Differentially abundant genera among the Control, DSS, and Br-H groups. ^*^P < 0.05. In panel I, A, B, C, and J represent the Control, DSS, DEX, and Br-H groups, respectively.

**Figure 5:**
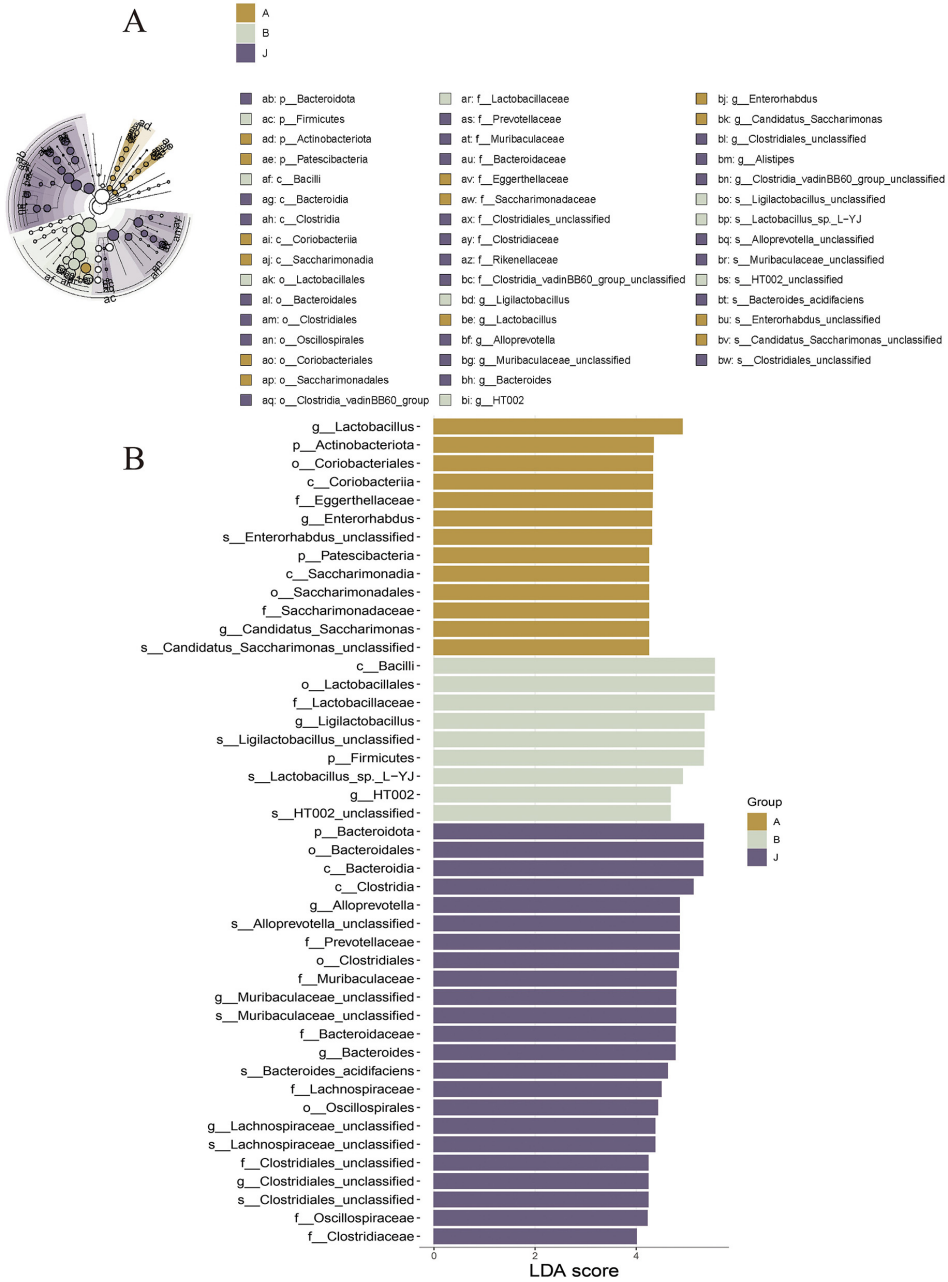
Differential microbial features identified by LEfSe analysis (LDA>3.0; *P* < 0.05). **(A)** Cladogram. Concentric rings from the center outward represent the taxonomic levels of kingdom, phylum, class, order, family, genus, and species. Each node denotes a taxon at the corresponding level, and node size is proportional to its relative abundance. Uncolored nodes indicate taxa without significant differences among groups, whereas yellow or green nodes indicate significantly different taxa; the color corresponds to the group with higher relative abundance. **(B)** Histogram of LDA scores. Bars represent statistically significant biomarkers; bar color indicates the group in which the taxon is more abundant, and bar length reflects the effect size of the intergroup difference. In panel I, A, B, and J represent the Control, DSS, and Br-H groups, respectively.

Phylum-level analysis showed that, compared with the Control group, the DSS group exhibited an increased relative abundance of *Firmicutes* and a decreased relative abundance of *Bacteroidetes*. Compared with the DSS group, both the Br-H and DEX groups showed a decreased relative abundance of Firmicutes and an increased relative abundance of *Bacteroidetes* (Figure 4A). Genus-level analysis further revealed that, in the DSS group, the relative abundances of genera such as *Ligilactobacillus, Lactobacillus*, and *HT002* increased, whereas those of *Bacteroides, Alloprevotella*, and *Lachnospiraceae_unclassified* decreased. These changes were partially reversed by Br-H and DEX treatment (Figure 4B). Subsequent analysis of differentially abundant genera showed that, in the DSS group, the relative abundances of genera previously reported to be associated with intestinal homeostasis and inflammatory regulation, including *Alloprevotella, Lachnospiraceae_unclassified*, and *Alistipes*, were markedly decreased (*P* < 0.05) (Bénard et al., 2025; Zhang et al., 2021; Liu et al., 2020; Lin et al., 2025), whereas the relative abundance of DSS-enriched genera, such as *HT002*, was significantly increased (*P* < 0.05). Following Br-H treatment, the relative abundances of *Alloprevotella, Lachnospiraceae_unclassified*, and *Alistipes* significantly increased, while the enrichment of *HT002* and other genera was reduced (*P* < 0.05; Figure 4C). Furthermore, LEfSe analysis revealed that the DSS group was primarily enriched in *Ligilactobacillus* and *HT002*, whereas the Br-H group was mainly enriched in *Alloprevotella* and *Lachnospiraceae_unclassified* (*P* < 0.05; Figures 5A, B). In summary, Br-H modulated DSS-induced gut microbiota dysbiosis by promoting the recovery of genera associated with intestinal homeostasis and reducing DSS-enriched genera, suggesting an improvement in intestinal microbial composition in mice with DSS-induced acute UC-like colitis.

### 3.5 Gut microbiota changes are correlated with inflammatory responses and intestinal mucosal barrier indicators in DSS-induced acute UC-like colitis

**Figure 6:**
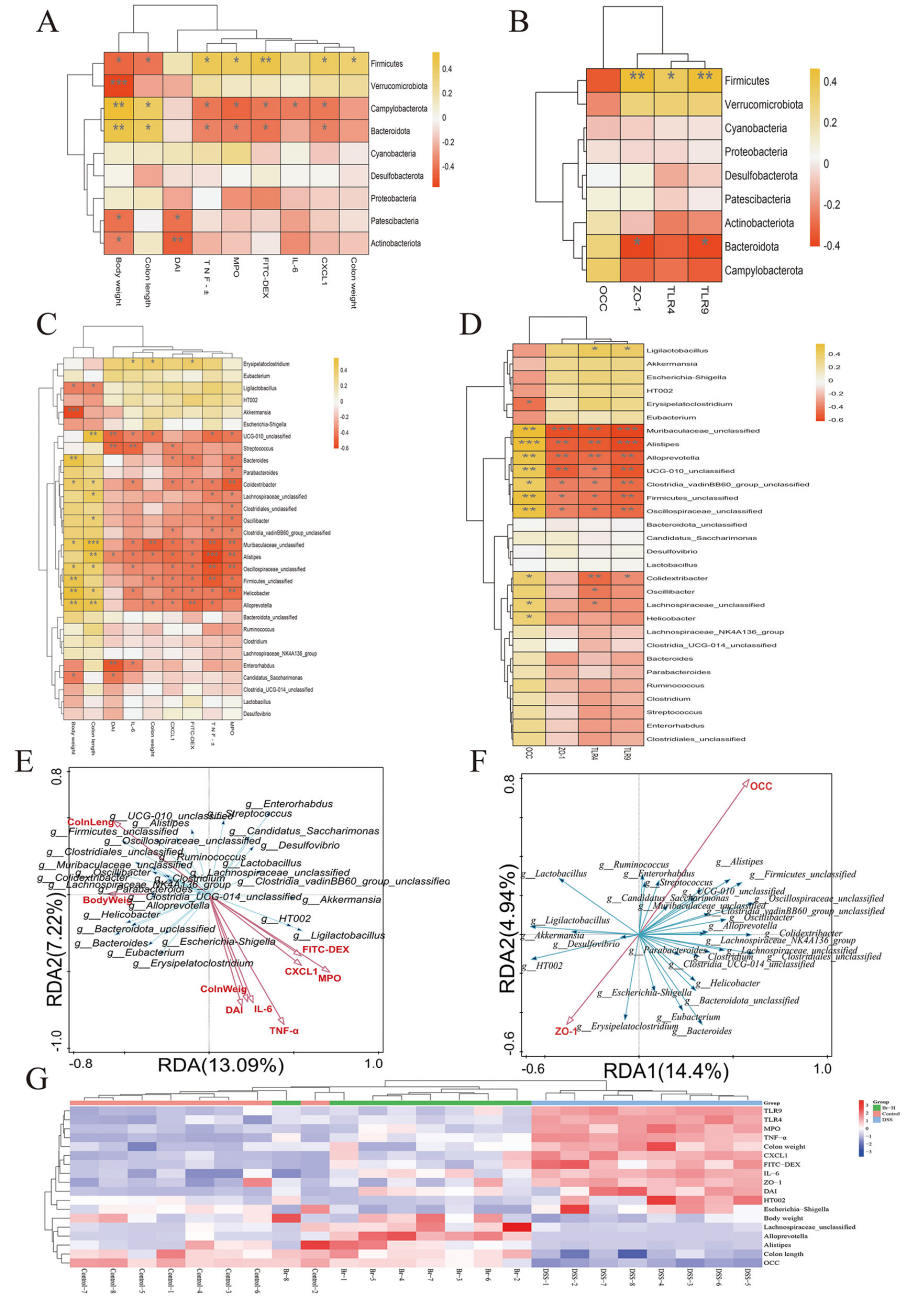
Correlation analysis between the gut microbiota and pathological indices. **(A)** Correlation analysis between phylum-level gut microbiota and pathological indices; **(B)** Correlation analysis between phylum-level gut microbiota and TLR4, TLR9, OCC, and ZO-1; **(C)** Correlation analysis between genus-level gut microbiota and pathological indices; **(D)** Correlation analysis between genus-level gut microbiota and TLR4, TLR9, OCC, and ZO-1; **(E)** Redundancy analysis (RDA) of genus-level gut microbiota and pathological indices; **(F)** RDA of genus-level gut microbiota in relation to TLR4, TLR9, OCC, and ZO-1; **(G)** Heatmap showing the correlations between the assessed indices and the relative abundances of *Alloprevotella, Lachnospiraceae_unclassified, Alistipes, HT002*, and *Escherichia-Shigella* across samples from the Control, DSS, and Br-H groups. ***P < 0.001, **P < 0.01, *P < 0.05.

Phylum-level correlation analysis revealed that *Firmicutes* was positively correlated with DAI, FITC-dextran levels, TLR4/TLR9 protein expression, and proinflammatory mediators, including IL-6, MPO, CXCL-1, and TNF-α, while it was negatively correlated with body weight, colon length, and tight junction protein levels. *Bacteroidetes* exhibited the opposite correlation pattern (Figures 6A, B). Genus-level correlation analysis further revealed that *HT002* was positively correlated with inflammation-related indicators and negatively correlated with body weight, colon length, and barrier function-related indicators. Conversely, *Alloprevotella, Lachnospiraceae_unclassified*, and *Alistipes* were positively correlated with body weight, colon length, and barrier function-related indicators, while negatively correlated with inflammation-related indicators (Figures 6C, D). Redundancy analysis further visualized the overall relationships between bacterial genera and multiple phenotypic indicators. Genera such as *Alloprevotella, Lachnospiraceae_unclassified*, and *Alistipes* formed acute angles with body weight, colon length, and TJ protein indicators. In contrast, HT002 tended to cluster with vectors representing disease activity and inflammation-related indicators (Figures 6E, F). Cluster analysis of the three sample groups revealed that DSS group samples primarily clustered with proinflammatory indicators, whereas Br-H group samples showed a stronger association with body weight, colon length, and barrier function-related indicators (Figure 6G). In summary, changes in gut microbiota structure were closely associated with inflammatory responses and intestinal mucosal barrier-related indicators in mice with DSS-induced acute UC-like colitis.

### 3.6 Br modulates gut metabolome profiles and related metabolic pathways in mice with DSS-induced acute UC-like colitis

**Figure 7:**
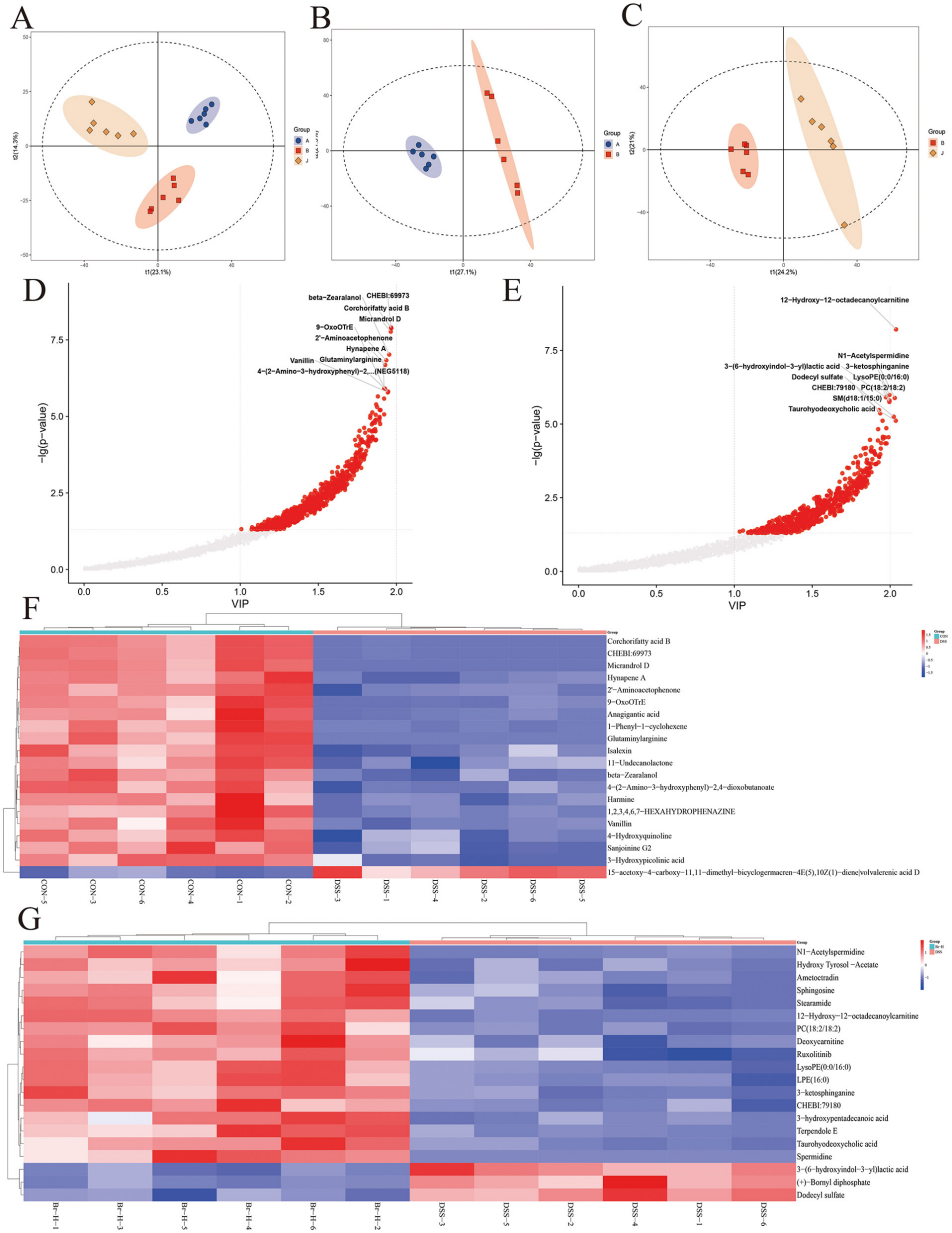
Gut metabolomics analysis (*n* = 6). **(A)** OPLS-DA score plot for the Control, DSS, and Br-H groups. **(B)** OPLS-DA score plot for the Control and DSS groups. **(C)** OPLS-DA score plot for the DSS and Br-H groups. **(D)** VIP–P-value scatter plot comparing the Control and DSS groups. **(E)** VIP–P-value scatter plot comparing the DSS and Br-H groups. **(F)** Clustered heatmap of the top 20 differential metabolites between the Control and DSS groups. **(G)** Clustered heatmap of the top 20 differential metabolites between the DSS and Br-H groups. In panel I, A, B, and J represent the Control, DSS, and Br-H groups, respectively.

OPLS-DA analysis revealed distinct metabolic profiles among the Control, DSS, and Br-H groups, with clear separation between groups (Figures 7A–C). To further identify metabolite features contributing to intergroup discrimination, VIP–*P*-value scatter plots were generated based on variable importance in projection values and statistical significance. These plots showed that DSS treatment induced marked metabolic alterations compared with the Control group, whereas Br-H treatment altered the DSS-associated metabolic profile (Figures 7D, E). Cluster analysis of the top 20 differential metabolite features revealed clear separation among groups with good intragroup clustering, supporting the reproducibility and intergroup discrimination of the metabolomic data (Figures 7F, G).

**Figure 8:**
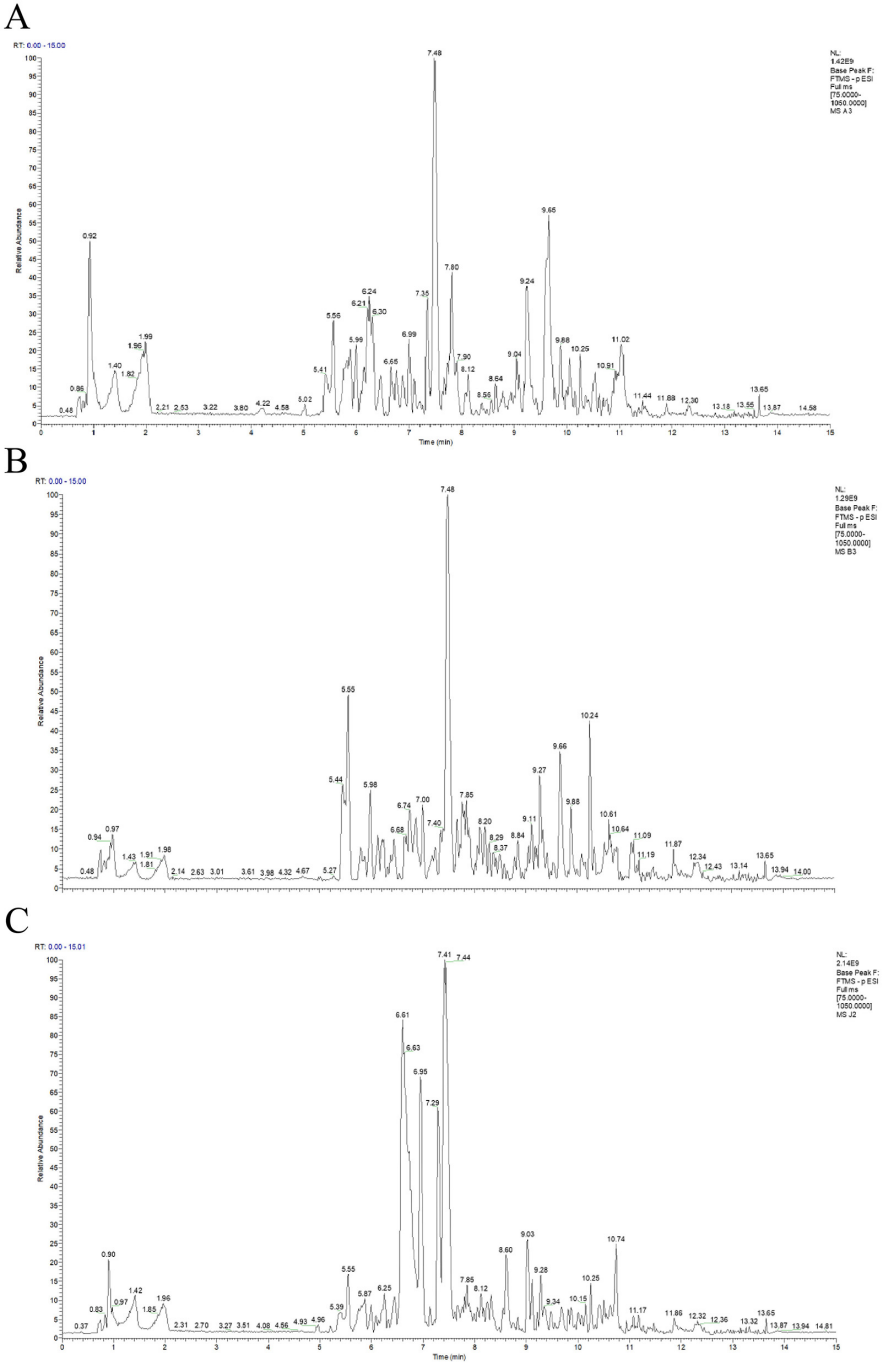
Representative UHPLC–MS/MS chromatograms: **(A)** Control, **(B)** DSS, **(C)** Br-H.

**Figure 9:**
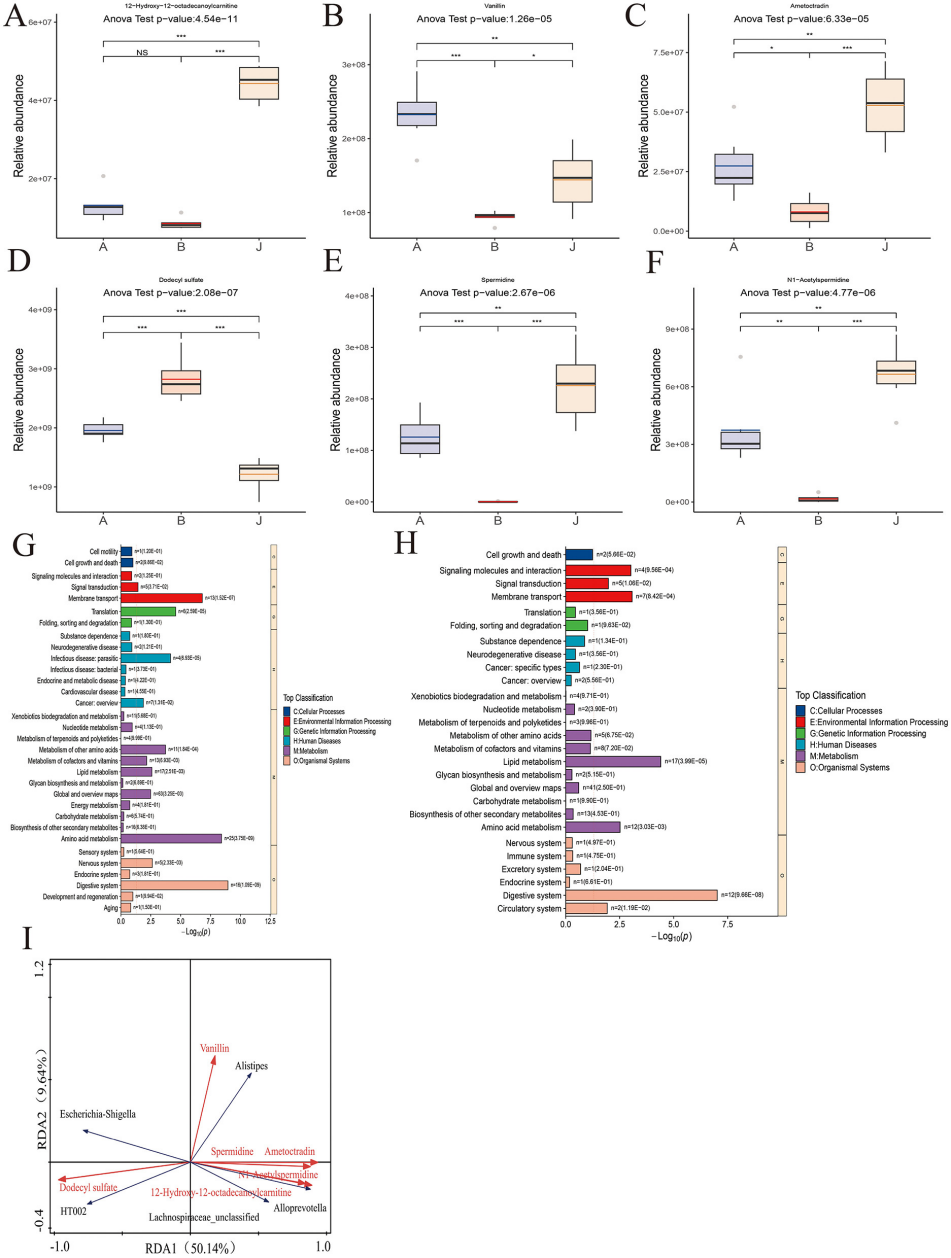
Shared differential metabolites and RDA-based correlation analysis. **(A–F)** Scatter plots of the shared differential metabolites among the Control, DSS, and Br-H groups; **(G)** KEGG pathway enrichment analysis of differential metabolites between the Control and DSS groups; **(H)** KEGG pathway enrichment analysis of differential metabolites between the DSS and Br-H groups; **(I)** RDA plot showing the associations between the shared differential metabolites and the genera *Alloprevotella, Lachnospiraceae_unclassified, Alistipes, HT002*, and *Escherichia-Shigella*. ***P < 0.001, **P < 0.01, *P < 0.05. In panel I, A, B, and J represent the Control, DSS, and Br-H groups, respectively.

Figure 8 further presents the UPLC-MS/MS spectra of the three groups. Further comparison of the metabolic profiles among the three groups led to the selection of six common candidate metabolite features, which were annotated as 12-Hydroxy-12-octadecanoylcarnitine, Vanillin, Ametoctradin, Dodecyl sulfate, Spermidine, and N1-Acetylspermidine (Table 1). Abundance analysis showed that, compared with the Control group, the abundance of a metabolite feature putatively annotated as Dodecyl sulfate was increased in the DSS group (*P* < 0.05). However, because Dodecyl sulfate is not generally considered a typical endogenous intestinal metabolite or a well-established gut microbiota-derived metabolite in mice, this annotation should be interpreted with caution. The relative abundances of several metabolite features annotated as 12-Hydroxy-12-octadecanoylcarnitine, Vanillin, Ametoctradin, Spermidine, and N1-Acetylspermidine were significantly increased after Br-H treatment (Figures 9A–F, *P* < 0.05). These annotations were based on database matching in non-targeted metabolomic analysis and should be further validated using authentic standards and targeted quantitative assays. KEGG enrichment analysis revealed that, compared with the Control group, differential metabolites in the DSS group were primarily enriched in pathways related to amino acid metabolism. In contrast, compared with the DSS group, differential metabolites in the Br-H group were predominantly enriched in pathways associated with lipid metabolism (Figures 9G, H). Further correlation analysis revealed that *Alloprevotella, Lachnospiraceae_unclassified*, and *Alistipes* were positively correlated with 12-Hydroxy-12-octadecanoylcarnitine, Vanillin, Ametoctradin, Spermidine, and N1-Acetylspermidine, while *HT002* and *Escherichia-Shigella* were positively correlated with Dodecyl sulfate (Figure 9I; Table 2). These findings suggest that Br-H treatment was associated with changes in DSS-induced intestinal metabolic alterations and microbiota-related metabolic profiles in mice with DSS-induced acute UC-like colitis.

**Table 1:** Differentially expressed metabolites in the Control and DSS and Br-H group.

Metabolite name	m/z	*P*	VIP	Trend	
				Control vs DSS	DSS vs Br-H
12-Hydroxy-12-octadecanoylcarnitine	444.37	0.00000000005	1.619373828	DOWN	DOWN
Vanillin	153.05	0.00001258613	1.309967016	DOWN	DOWN
Ametoctradin	276.22	0.00006328053	1.033326911	DOWN	DOWN
Dodecyl sulfate	265.15	0.00000020779	0.858811055	UP	UP
Spermidine	146.17	0.00000267419	0.803952087	DOWN	DOWN
N1-Acetylspermidine	188.18	0.00000476795	0.80244937	DOWN	DOWN

**Table 2:** Correlations between gut microbiota and serum metabolites.

Genus	Metabolites	*R*	*P*
*HT002*	12-Hydroxy-12-octadecanoylcarnitine	−0.5804	0.0116
	Vanillin	−0.4172	0.0850
	Ametoctradin	−0.6387	0.0043
	Dodecyl sulfate	0.7987	0.0001
	Spermidine	−0.7189	0.0008
	N1-Acetylspermidine	−0.6673	0.0025
*Alloprevotella*	12-Hydroxy-12-octadecanoylcarnitine	0.8969	0.0001
	Vanillin	−0.1202	0.6346
	Ametoctradin	0.7197	0.0008
	Dodecyl sulfate	−0.7852	0.0001
	Spermidine	0.8265	0.0001
	N1-Acetylspermidine	0.6681	0.0024
*Lachnospiraceae_ unclassified*	12-Hydroxy-12-octadecanoylcarnitine	0.4593	0.0552
	Vanillin	0.0245	0.9230
	Ametoctradin	0.5794	0.0117
	Dodecyl sulfate	−0.5959	0.0091
	Spermidine	0.5154	0.0286
	N1-Acetylspermidine	0.5697	0.0136
*Alistipes*	12-Hydroxy-12-octadecanoylcarnitine	−0.7485	0.0004
	Vanillin	−0.0721	0.7761
	Ametoctradin	−0.7421	0.0004
	Dodecyl sulfate	0.7373	0.0005
	Spermidine	−0.7615	0.0002
	N1-Acetylspermidine	−0.7336	0.0005
*Escherichia-Shigella*	12-Hydroxy-12-octadecanoylcarnitine	0.1379	0.5853
	Vanillin	−0.4063	0.0943
	Ametoctradin	−0.0245	0.9231
	Dodecyl sulfate	0.1728	0.4928
	Spermidine	−0.1599	0.5262
	N1-Acetylspermidine	−0.0519	0.8378

## 4 Discussion

The acute DSS-induced UC-like colitis model was used in the present study to investigate whether Br could alleviate chemically induced intestinal inflammation and barrier dysfunction. DSS exposure induces acute colonic epithelial injury, increased intestinal permeability, inflammatory cell infiltration, colon shortening, and elevated proinflammatory cytokine production. These pathological changes resemble several features of UC, particularly mucosal inflammation and epithelial barrier disruption, making this model commonly used for evaluating intestinal inflammatory injury and mucosal barrier dysfunction (Chassaing et al., 2014; Eichele and Kharbanda, 2017). Thus, the DSS-induced acute UC-like colitis model is suitable for the preliminary assessment of the anti-inflammatory and barrier-protective effects of Br (Yang and Merlin, 2024). Nevertheless, the results should be interpreted as evidence from a DSS-induced acute colitis-like injury model in mice (Yang and Merlin, 2024). Within this experimental context, this study demonstrates that Br significantly ameliorates DSS-induced acute colitis-like phenotypes in mice, including weight loss, elevated DAI scores, colon shortening, increased intestinal permeability, and histopathological damage. These effects were accompanied by reduced levels of IL-6, MPO, CXCL-1, and TNF-α, decreased TLR4 and TLR9 expression, and restored OCC and ZO-1 expression, suggesting that Br may exert protective effects against DSS-induced intestinal inflammation and barrier dysfunction.

Previous studies indicate that excessive TLR4 activation induces downstream MyD88/NF-κB signaling, thereby triggering the release of proinflammatory mediators. In contrast, TLR9 exhibits dual roles in the gut: moderate activation supports immune tolerance and mucosal repair, whereas abnormal upregulation during inflammation often indicates persistent activation of innate immunity. In the present study, Br treatment downregulated abnormal TLR4/TLR9 expression and restored the expression of barrier-related proteins, suggesting that the protective effects of Br may be partly associated with the modulation of innate immune responses and epithelial barrier integrity. However, because downstream molecules such as MyD88 and NF-κB were not examined, further studies are needed to clarify whether this pathway is directly involved.

Gut microbiota analysis further suggested that microbial modulation may be associated with the effects of Br in DSS-induced acute UC-like colitis. DSS treatment led to decreased α-diversity and reduced ASV abundance, whereas Br intervention improved these alterations and promoted a partial recovery of microbial composition. Previous studies have shown that gut microbiota dysbiosis and reduced diversity are key features of IBD, including UC, and that these changes are closely associated with disease activity and persistent inflammation (Matsuoka and Kanai, 2015; Stange and Schroeder, 2019). Although DSS treatment altered the relative abundances of *Firmicutes* and *Bacteroidetes* in the present study, phylum-level changes alone may not fully reflect the complexity of gut microbiota dysbiosis. Therefore, the following discussion focuses primarily on bacterial taxa at lower taxonomic levels. In accordance with these findings, Br treatment increased the relative abundances of potentially beneficial taxa, including *Alloprevotella, Lachnospiraceae_unclassified*, and *Alistipes*, while reducing the relative abundances of genera such as *HT002* and *Escherichia-Shigella*. *Lachnospiraceae_unclassified* has been associated with short-chain fatty acid production and the maintenance of mucosal homeostasis, whereas *Alloprevotella* and *Alistipes* have also been associated with reduced inflammation and improved barrier function (Bénard et al., 2025; Zhang et al., 2021; Liu et al., 2020; Lin et al., 2025). Correlation analysis further revealed that these potentially beneficial bacteria were positively correlated with body weight, colon length, and barrier function-related indicators, while negatively correlated with inflammation-related indicators and disease activity markers. Conversely, *Escherichia-Shigella* is often enriched in IBD, and its increased abundance has been associated with gut dysbiosis and aggravated mucosal inflammation (He et al., 2021; Hu et al., 2022). The increased relative abundance of *HT002* in the DSS group also suggests its possible association with intestinal inflammation in this model. Taken together, these findings suggest that Br treatment was associated with partial restoration of DSS-disrupted gut microbial composition in mice with acute UC-like colitis.

Metabolomics findings further suggest that the effects of Br on DSS-induced acute UC-like colitis may be accompanied by alterations in the intestinal metabolic profile. In this study, the relative abundances of several metabolite features annotated as 12-Hydroxy-12-octadecanoylcarnitine, Vanillin, Ametoctradin, Spermidine, and N1-Acetylspermidine were increased after Br-H treatment, and these features were positively correlated with *Alloprevotella, Lachnospiraceae_unclassified*, and *Alistipes*. In contrast, a feature putatively annotated as Dodecyl sulfate was elevated in the DSS group and was positively correlated with *HT002* and *Escherichia-Shigella*. Previous studies have reported that Vanillin and Spermidine exert anti-inflammatory and intestinal barrier-protective effects in experimental colitis (Wu et al., 2009; Lv et al., 2025; Yan et al., 2023), whereas N1-Acetylspermidine may reflect alterations in polyamine metabolism, which may be associated with host–microbiota co-metabolic processes (Pegg, 2008; Nakamura et al., 2021). Although 12-Hydroxy-12-octadecanoylcarnitine has not been directly linked to IBD or UC, changes in this acylcarnitine-related feature may reflect alterations in lipid and energy-related metabolic processes after Br-H intervention. Notably, Ametoctradin and Dodecyl sulfate are not generally considered typical endogenous intestinal metabolites or well-established gut microbiota-derived metabolites. Ametoctradin is more commonly known as an exogenous fungicide-related compound, whereas Dodecyl sulfate/sodium dodecyl sulfate is commonly described as an anionic surfactant or detergent-related compound. Therefore, the signals annotated as Ametoctradin and Dodecyl sulfate in the present non-targeted metabolomic analysis should be interpreted with caution. These annotations may reflect exogenous/background exposure, sample processing-related contamination, DSS-related experimental interference, or limitations of database-based metabolite annotation rather than true endogenous metabolic changes. Further targeted metabolomics, authentic standard verification, and quantitative analyses are required to confirm their chemical identities, sources, and biological relevance. KEGG analysis revealed that DSS-induced differential metabolites were primarily enriched in amino acid metabolism-related pathways, whereas Br-H-associated differential metabolites were predominantly enriched in lipid metabolism-related pathways. Previous studies indicate that IBD, including UC, is frequently accompanied by disturbances in amino acid metabolism, which are closely associated with inflammatory stress, immune activation, and dysbiosis. In addition, abnormalities in lipid metabolism are related to energy supply, inflammatory regulation, and barrier repair (Upadhyay et al., 2023; Di Ciaula et al., 2023). Thus, DSS-associated changes in amino acid metabolism pathways may reflect metabolic disturbance under intestinal inflammatory conditions. In contrast, the enrichment of lipid metabolism pathways following Br-H intervention may be associated with changes in intestinal lipid metabolism and energy-related processes. However, these metabolomic findings are mainly associative and require further validation.

In summary, Br may alleviate DSS-induced acute UC-like colitis injury through coordinated regulation of inflammatory responses, intestinal mucosal barrier function, gut microbiota composition, and metabolite profiles. However, several limitations should be considered. First, although the DSS-induced acute colitis model resembles several pathological features of UC, particularly epithelial injury and mucosal inflammation, it mainly reflects chemically induced intestinal inflammation and barrier injury and cannot fully reproduce the chronic, relapsing, immune-mediated, and heterogeneous features of human UC or the broader spectrum of IBD. Thus, the present findings should be interpreted as preclinical evidence from a DSS-induced acute UC-like colitis model in mice, rather than as direct evidence of clinical efficacy in human UC or IBD. Second, the microbiota and metabolomics results are mainly correlative, and the causal roles of key bacterial genera and candidate metabolite features remain to be confirmed. Third, although TLR4/TLR9 expression was examined, downstream molecules such as MyD88 and NF-κB were not further assessed. In addition, the sources and biological relevance of some putatively annotated metabolites, especially Ametoctradin and Dodecyl sulfate, require cautious interpretation. Future studies using chronic or recurrent colitis models, immune-mediated models, fecal microbiota transplantation, germ-free or antibiotic-depleted models, targeted metabolomics, and authentic standard validation are needed to further clarify the causal relationships among Br, gut microbiota, metabolites, and host responses. Further identification of the major active constituents of Br will also help evaluate its potential as a natural product or functional food for UC-related intestinal inflammation.

## 5 Conclusion

This study demonstrates that Br water extract alleviates DSS-induced acute UC-like colitis injury in mice, as evidenced by attenuated body weight loss, reduced disease activity index scores, restored colon length, decreased intestinal hyperpermeability, and diminished histopathological damage in colonic tissue. Br also reduced the levels of inflammatory markers, including IL-6, MPO, CXCL-1, and TNF-α. In addition, Br downregulated TLR4 and TLR9 expression while upregulating OCC and ZO-1 expression, suggesting that it contributed to the suppression of inflammatory responses and the improvement of intestinal mucosal barrier function. Further analyses revealed that Br improved DSS-induced gut microbiota dysbiosis by promoting the recovery of potentially beneficial bacteria such as *Alloprevotella, Lachnospiraceae_unclassified*, and *Alistipes*, while modulating metabolic disturbances associated with inflammation and barrier function. In summary, Br alleviated DSS-induced acute UC-like colitis injury by modulating TLR4/TLR9-related inflammatory responses, improving intestinal mucosal barrier function, and reshaping gut microbiota composition and metabolic profiles, thereby supporting its potential value as a candidate natural product for further investigation in UC-related intestinal inflammation.

## Data Availability

The raw data generated in this study can be found in the NCBI (https://www.ncbi.nlm.nih.gov), accession PRJNA1476925.
